# Head-to-Head Comparison of Different Software Solutions for AVC Quantification Using Contrast-Enhanced MDCT

**DOI:** 10.3390/jcm10173970

**Published:** 2021-09-02

**Authors:** Ruben Evertz, Sebastian Hub, Sören J. Backhaus, Torben Lange, Karl Toischer, Johannes T. Kowallick, Gerd Hasenfuß, Andreas Schuster

**Affiliations:** 1 Department of Cardiology and Pneumology, University Medical Center Goettingen (UMG), 37075 Goettingen, Germany; ruben.evertz@med.uni-goettingen.de (R.E.); s.hub@stud.uni-goettingen.de (S.H.); soeren.backhaus@med.uni-goettingen.de (S.J.B.); torben.lange@med.uni-goettingen.de (T.L.); ktoischer@med.uni-goettingen.de (K.T.); hasenfus@med.uni-goettingen.de (G.H.); 2 German Center for Cardiovascular Research (DZHK), 37099 Göttingen, Germany; johannes.kowallick@med.uni-goettingen.de; 3 Department of Diagnostic & Interventional Radiology, University Medical Center Goettingen (UMG), 37075 Goettingen, Germany

**Keywords:** aortic stenosis, aortic valve calcification, contrast-enhanced MDCT, inter-vendor variability

## Abstract

Aortic valve calcification (AVC) in aortic stenosis patients has diagnostic and prognostic implications. Little is known about the interchangeability of AVC obtained from different multidetector computed tomography (MDCT) software solutions. Contrast-enhanced MDCT data sets of 50 randomly selected aortic stenosis patients were analysed using three different software vendors (3Mensio, CVI42, Syngo.Via). A subset of 10 patients were analysed twice for the estimation of intra-observer variability. Intra- and inter-observer variability were determined using the ICC reliability method, Bland-Altman analysis and coefficients of variation. No differences were revealed between the software solutions in the AVC calculations (3Mensio 941 ± 623, Syngo.Via 948 mm^3^ ± 655, CVI42 941 ± 637; *p* = 0.455). The best inter-vendor agreement was found between the CVI42 and the Syngo.Via (ICC 0.997 (CI 0.995–0.998)), followed by the 3Mensio and the CVI42 (ICC 0.996 (CI 0.922–0.998)), and the 3Mensio and the Syngo.Via (ICC 0.992 (CI 0.986–0.995)). There was excellent intra- (3Mensio: ICC 0.999 (0.995–1.000); CVI42: ICC 1.000 (0.999–1.000); Syngo.Via: ICC 0.998 (0.993–1.000)) and inter-observer variability (3Mensio: ICC 1.000 (0.999–1.000); CVI42: ICC 1.000 (1.000–1.000); Syngo.Via: ICC 0.996 (0.985–0.999)) for all software types. Contrast-enhanced MDCT-derived AVC scores are interchangeable between and reproducible within different commercially available software solutions. This is important since sufficient reproducibility, interchangeability and valid results represent prerequisites for accurate TAVR planning and its widespread clinical use.

## 1. Introduction

Aortic stenosis (AS) is the most common valvular heart disease in the elderly population in Europe and North America [1,2]. Whilst historically surgery was the only therapeutic approach, treatment options have changed in the last 19 years. On 16 April 2002, the first transcatheter aortic valve replacement (TAVR) was performed [3]. Although TAVR was initially only performed in patients with very high risk, TAVR has since become the standard treatment for the elderly population and can be safely recommended to low-risk patient populations [4,5]. In 2018, almost 21,000 TAVR procedures were performed in Germany and there has been a steady increase in the number of interventions over the last 10 years [6]. Depending on the transvalvular flow and the transvalvular pressure gradient, different AS subtypes can be distinguished on the basis of echocardiography. Data from several studies suggest a prevalence of (paradoxical) low-flow low-gradient AS in up to 35% of cases [7,8,9,10]. In the absence of a high-gradient situation the diagnostic workup is challenging and further techniques beside the initial resting echocardiography are needed. In low-flow low-gradient AS a dobutamine stress echocardiography is performed to exclude pseudo-severe AS, whereas in a paradoxical low-flow low-gradient situation or in a low-flow low-gradient situation without a contractile reserve, multidetector computed tomography (MDCT) is required to assess the severity of AS, using the estimated aortic valve calcification (AVC) [1,11]. Furthermore, besides its diagnostic capabilities, MDCT also carries prognostic implications. High amounts of AVC are associated with an increased mortality rate in patients with severe AS [12,13,14]. Besides its diagnostic and prognostic role, MDCT is also mandatory for planning a TAVR procedure, in which a contrast-enhanced scan is used to gather information about the vascular approach and to select the right valve size. 

For the quantification of AVC, a native scan is used to determine the calcium score expressed as Agatston units. Nevertheless, an additional native CT scan carries the burden of increased radiation exposure and there is data to suggest that accurate quantification of the AVC load can be obtained from contrast-enhanced studies [15,16]. However, multiple software solutions are used for TAVR planning and AVC quantification and neither clear recommendations regarding their use nor interpretations of the derived data exist. Considering that the widespread clinical use of AVC quantification is highly desirable and important, the pre-requisites for the achievement of this goal are to ensure that the assessments are reproducible and comparable with a high amount of inter-vendor agreement. 

Therefore, we aimed to compare three different software solutions in regards to their reproducibility and interchangeability of AVC measurements on contrast enhanced MDCT scans.

## 2. Materials and Methods

Fifty patients, who underwent TAVR between January 2017 and October 2018, were enrolled in the current study. Written informed consent was obtained from all patients and the study was conducted according to the principles of the Helsinki Declaration.

### 2.1. Echocardiography

In all patients the presence of severe AS was confirmed by transthoracic echocardiography (TTE) and the AS was classified according to current guidelines [1]. TTE was performed using either a Philips ie33 or a Philips Epiq7 system. The post-processing and severity measurements were performed by a physician specialising in TTE.

### 2.2. Multidetector Computed Tomography

**Figure 1:**
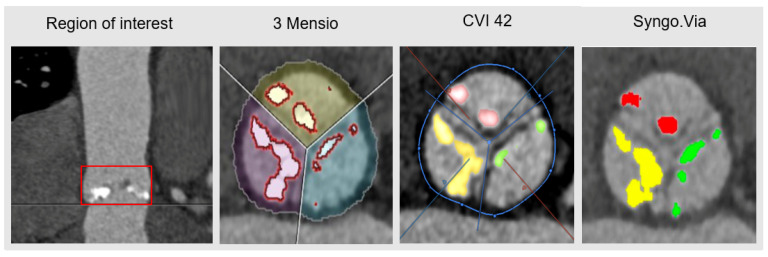
Visualisation of Aortic Valve Calcification. A representative illustration of the region of interest (coronary view) and of the aortic valve (transversal view), using the different software types. Aortic valve calcification is marked and differently colour coded for the aortic cusps, respectively.

Contrast-enhanced MDCT scans were performed with a dual-source CT scanner (SOMATOM Force, Siemens Healthcare GmbH, Erlangen, Germany), using a prospectively ECG-triggered high-pitch spiral acquisition mode, extending from the clavicles to the femoral heads. CT angiography was performed with bolus tracking in the descending aorta, using a 80 mL contrast agent bolus (Iomeron 350, Bracco Imaging, Konstanz, Germany) at a flow rate of 4 mL/sec followed by a 40 mL saline chaser at the same flow rate. Scan parameters were as follows: 2 × 192 × 0.6 mm collimation, 250 ms rotation time, pitch of 3.2 and automated tube current adaption. A small field of view data set with medium soft convolution kernel (Siemens Bv36), 0.75 mm slice thickness was generated for the assessment of the aortic annulus, root, and valve morphology and dimensions. Aortic valve calcification was defined as calcification within the valve leaflets, aortic annulus or aortic wall up to the sinotubular junction. The calcification of the coronary arteries was excluded from the region of interest (Figure 1). The calcium score was expressed as mm^3^ [11]. All data were analysed using commercially available software provided by: 1. “3 Mensio” (3 Mensio, Structural Heart, V9.1, Pie Medical Imaging, Maastricht, Netherlands); 2. “CVI 42” (CVI 42, V5.11.3, Circle Cardiovascular Imaging Inc., Calgary, Canada); and 3. “Syngo.Via” (Syngo.Via, Version VB50B_HF01, Siemens Healthcare GmbH, Erlangen, Germany) (Figure 1). An individual Hounsfield Units (HU) threshold was used to discriminate between the calcium and contrast mediums, which was in line with current research [15,16,17,18]. For the individual adjustment of HU, a visual adjustment was made based on an empirical threshold (HU 550), as has been proposed by Ludwig et al. [16].

### 2.3. Statistical Analysis

The statistical analysis was conducted using Microsoft Excel and IBM SPSS Statistics version 26 for Windows. Data are expressed as mean ± standard deviations. Normal distribution was tested using the Shapiro-Wilk test. Non-parametric data were compared using the Wilcoxon and Friedmann tests, as appropriate. For between-group comparisons in parametric data t- or ANOVA testing was performed, as appropriate. The *p*-values provided are two-sided; an alpha level of ≤0.05 was considered statistically significant.

A subset of 10 randomly selected patients was repeatedly analysed after at least 2 weeks to assess intra-observer variability. The analysis of a second skilled observer was used to assess inter-observer reproducibility. Intra- and inter-observer variability was quantified using the ICC reliability method, Bland-Altman analysis and coefficients of variation (CoV) [19]. CoV was defined as the standard deviation of the differences divided by the mean. ICC Reliability was scored as follows: excellent (>0.74), good (0.6–0.74), fair (0.4–0.59) and poor (<0.4), as previously defined [20].

## 3. Results

### 3.1. Patients’ Characteristics

The patients’ characteristics are displayed in Table 1. Approximately two-thirds of the patients were male. The mean age was 78 ± 6 years. The youngest patient was 61 and the oldest 92 years old. All patients were suffering from severe AS, which was diagnosed according to current guideline recommendations [1]. The patients were classified according to AS subtypes as follows: high-ejection-fraction high-gradient (HEFHG) AS 27 (54%), low-ejection-fraction high-gradient (LEFHG) AS 4 (8%), low-ejection-fraction low-gradient (LEFLG) AS 8 (16%) and paradoxical low-flow low-gradient (PLFLG) AS 11 (22%). The mean peak transvalvular velocity and the mean transvalvular pressure gradient were 4.1 ± 0.6 (2.9–5.5) and 40 ± 14 (16–68), respectively. 

The most common cardiovascular comorbidities were hypertension (86%) and coronary artery disease (80%), followed by diabetes (24%), atrial fibrillation (14.8%) and COPD (12%). 

### 3.2. AVC Quantification

**Figure 2:**
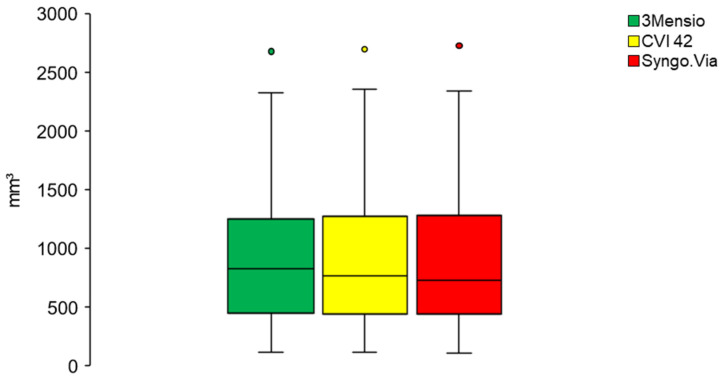
AVC calculation using three software solutions. A comparison of AVC measured in a cohort of 50 patients, using three different software types. There was no significant difference in AVC (Friedmann test for continuous data with deviations from normality; *p* = 0.455). AVC: aortic valve calcification.

**Figure 3:**
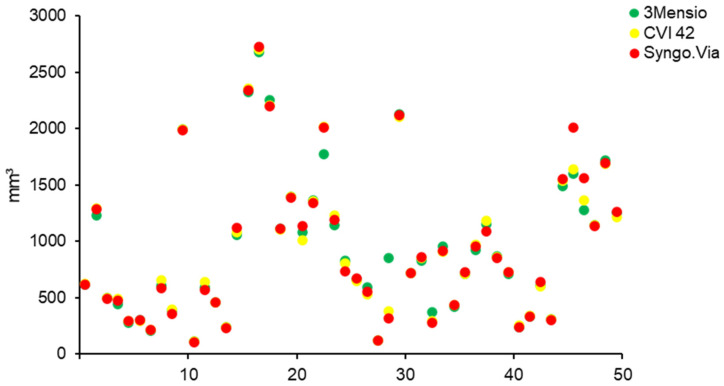
AVC measurements for each patient, using three different software solutions. A representation of the individual measurements of AVC for each patient using the three different software types. AVC: aortic valve calcification.

All measurements were performed using an individual HU threshold (591 ± 132 (450–950)). The degree of AVC was successfully measured in all patients using all software types. Mean calcium load did not differ between the used software types (*p* = 0.455) (Figure 2). On an individual patient basis, the differences were minimal, as shown in Figure 3. The highest amount of AVC was detected using the Syngo.Via (948 mm^3^ ± 655 (108–2724)), followed by the CVI 42 (941 ± 637 (117–2698)) and the 3 Mensio (941 ± 623 (117–2678)). 

### 3.3. Inter-Vendor Reproducibility

**Figure 4:**
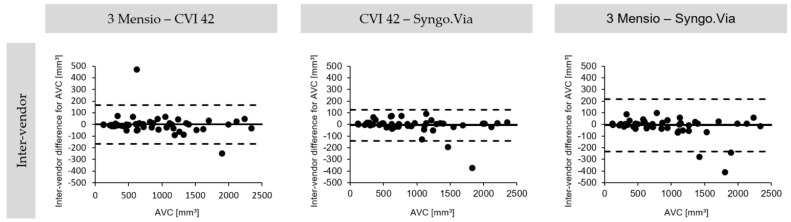
Bland-Altmann plots for inter-vendor agreement. The inter-vendor agreement on the degree of AVC. Bland-Altmann plots with limits of agreement (95% confidence intervals) demonstrate the inter-vendor reproducibility of MDCT-derived AVC. MDCT: multidetector computed tomography; AVC: aortic valve calcification.

Excellent inter-vendor agreement was seen when comparing all software types (Figure 4 and Table 2). The CVI 42 and the Snygo.Via (ICC 0.997 (CI 0.995–0.998), CoV 7.3%) showed the highest numerical agreement, followed by the 3 Mensio with the CVI (ICC 0.996 (CI 0.922–0.998), CoV 9%) and the 3 Mensio with the Syngo.Via (ICC 0.992 (CI 0.986–0.995), CoV 12.2%).

### 3.4. Intra-Vendor Reproducibility

**Figure 5:**
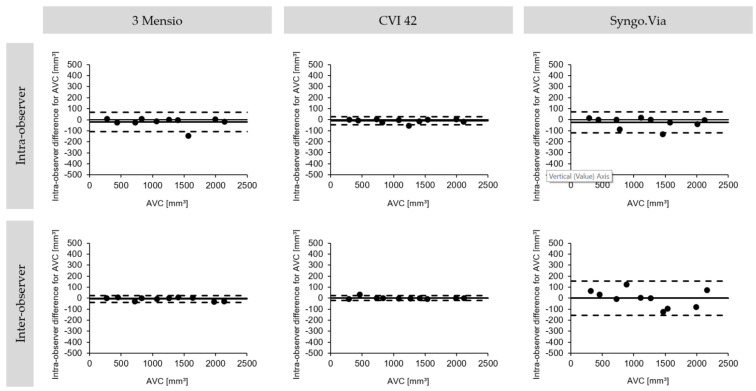
Bland-Altman plots for intra- and inter-observer agreement. The intra- and interobserver agreement on the degree of AVC. Bland-Altmann plots with limits of agreement (95% confidence intervals) demonstrate the reproducibility of MDCT-derived AVC using three different software solutions. The data for inter-observer reproducibility were derived by a second skilled observer. MDCT: multidetector computed tomography; AVC: aortic valve calcification.

**Figure 6:**
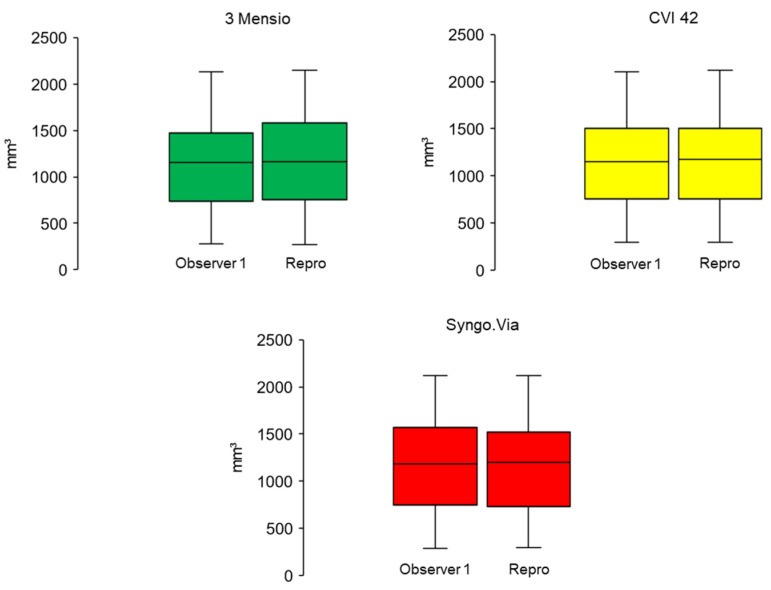
Intra-observer AVC measurements for the three different software solutions. An illustration of the AVC measured in the intra-observer setting. No significant differences were found. (*t*-Test for paired, normally distributed samples; 3 Meniso *p* = 0.209, CVI 42 *p* = 0.114, Syngo.Via *p* = 0.123). AVC: aortic valve calcification.

Intra- and inter-observer agreement was high for all the programs used. The best intra- and inter-observer reproducibility was found in the CVI 42, followed by the 3 Mensio and the Syngo.Via. Details are expressed in Figure 5 and Table 3. In the first analysis run, the mean AVC was 1159 mm^3^ ± 613 (294–2102), estimated by CVI 42. There was no significant difference compared to the repeated measurement, which showed a mean AVC of 1170 mm^3^ ± 614 (293–2118) (*p* = 0.114). Furthermore, no differences were found using the 3 Mensio, which showed a mean AVC of 1157 mm^3^ ± 620 (280–2131), compared to the second run, which showed a mean calcium load of 1177 mm^3^ ± 630 (270–2148) (*p* = 0.209). Likewise, the results of the first (1163 mm^3^ ± 621 (297–2122)) and the second measurement (1188 mm^3^ ± 632 (282–2124)) using Syngo.Via were not different (*p* = 0.123). Figure 6 provides a graphic illustration.

**Figure 7:**
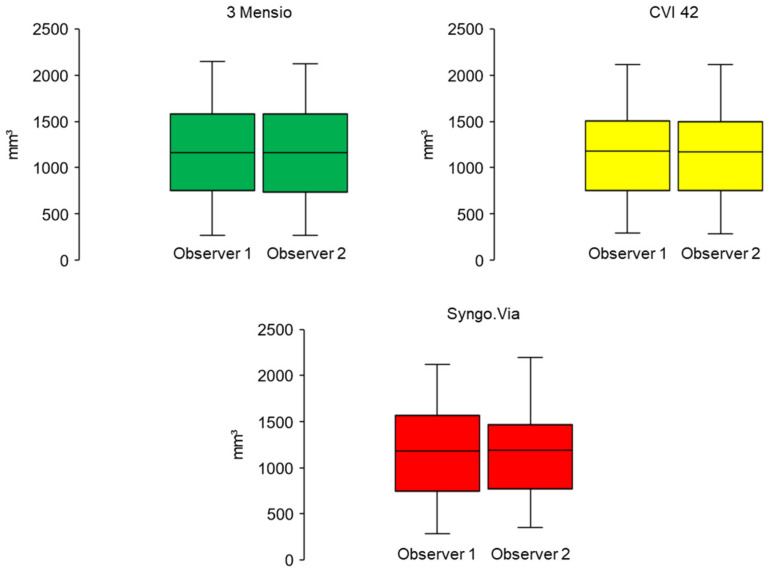
Inter-observer AVC measurements for the three different software solutions. An illustration of the AVC measured in the inter-observer setting. No significant differences were found. (*t*-Test for paired, normally distributed samples; 3 Meniso *p* = 0.197, CVI 42 *p* = 0.653, Scheme 0). AVC: aortic valve calcification.

The inter-observer comparison also showed no significant differences. The second trained operator measured an average AVC of 1171 mm^3^ ± 610 (288–2118) for CVI 42 (*p* = 0.653), 1170 mm^3^ ± 622 (270–21,199) with the 3 Mensio (*p* = 0.197), and 1189 mm^3^ ± 600 with the Syngo.Via (*p* = 0.980). Figure 7 provides a graphic illustration.

## 4. Discussion

The current study presents data of randomly selected TAVR patients undergoing planned contrast-enhanced MDCT and demonstrates the reproducibility and interchangeability data from three commercially available software types that are already widely distributed in clinical TAVR planning. 

The following notable findings should be considered. Firstly, all utilized software types provided accurate calcium quantification of contrast-enhanced TAVR planning scans. Secondly, there was interchangeability between the different software types, suggesting a sufficient level of accuracy for any given method in the clinical arena. Finally, while all software solutions showed excellent reproducibility, CVI 42 qualified as the most reproducible product, which may be considered if one had to choose a given software.

While the determination of AVC using native CT is an established procedure and has already been included in current guidelines [1], several studies have shown that a sufficient calcium quantification is also possible with contrast medium CT [15,16,21,22]. To our knowledge, this is the first study evaluating the differences in AVC estimation depending on the software used in contrast-enhanced MDCT-scans. 

Our results demonstrate high reproducibility within and between vendors. This suggests the feasibility of the widespread use of all the investigated solutions for AVC quantification. This is in opposition to other imaging tests, especially functional imaging tests. Echocardiography and cardiac magnetic resonance imaging (MRI) speckle tracking and feature tracking have been repeatedly shown to be susceptible to inaccuracies introduced by different software solutions, with significant differences in the determination of the global longitudinal strain, depending on the software used. Furthermore, speckle tracking results derived from different software types are not interchangeable [23,24]. Feature tracking is an analogous technique to speckle tracking but in contrast data are not resulting from echocardiography but from cardiac MRI [25]. MRI studies present different results for inter-vendor reproducibility depending on the software used [26,27]. Therefore, the software used must be taken into account when interpreting these data and interchangeability, which has complicated, widespread clinical applications, is not restricted. Conversely, we have demonstrated that MDCT AVC quantification is interchangeable between three commonly used analysis software solutions. Similar results regarding MDCT-derived aortic measurements (annulus diameter min/max, annulus perimeter, annulus area) prior to TAVR using different software vendors were observed by Baeßer et al. The authors also concluded that there was interchangeability between the used software solutions [28]. A possible explanation for the inter-vendor interchangeability of the AVC and aortic measurement data, compared to functional data coming from echocardiography and cardiac MRI speckle tracking and feature tracking, is the fact that calcium quantification and aortic measurements are generated from static images, while functional measurements are based on dynamic images, which are susceptible to through-plane motion. In addition, the region of interest in the determination of AVC is characterized by easily identifiable anatomical structures, which leads to more robust data even if vendors are using different algorithms and labelling methods for calcification quantification. However, the latter could be a possible explanation for the observed small and non-significant inter-vendor numerical differences. Furthermore, observer-induced inaccuracies in the performed measurements may also result in non-significant inter-vendor and intra-vendor differences, which can be attributed, for example, to an inaccurate definition of the region of interest. This may lead to the inclusion of extra aortic calcification within the analysis. Typical examples in this context are the inclusion of the LVOT if the annulus plane is moved towards the left ventricle and the inclusion of calcified coronary ostia.

Our findings allow the conclusion that measured AVC can be interpreted independently of the software used in contrast-enhanced MDCT scans. In 2019, Eberhard et al. described for the first time the inter-vendor interchangeability of AVC quantification in non-contrast-enhanced MDCTs [29], whereas our study confirmed that interchangeability was also present in contrast-enhanced MDCTs. Additionally, the observed transferability was also proven for Syngo.Via, which was not included in the study by Eberhard et al. Taken together, both studies demonstrate the feasibility of MDCT for the calcium quantification of the aortic valve, with likely significant clinical implications. These results coincide with data from non-contrast-enhanced coronary artery MDCTs, according to which the likelihood of coronary artery disease was estimated by determining the calcium load, which underscores the importance of calcium quantification as a pathological substrate [30,31].

As mentioned above, the interchangebility of MDCT that can be inferred from our results is particularly important in the diagnostic workup in the absence of a high transvalvular gradient or a high transvalvular velocity. Different studies showed that up to 35% of all cases of AS are attributed to the PLFLG subtype [7,8,9,10]. Additionally, registry data of the German aortic valve registry classified 11.7% of cases as LEFLG AS and 20.8% as PLFLG AS. Therefore, a significant number of AS patients require accurate AVC quantification before TAVR, highlighting the clinical importance of sufficient software analyses [32]. Recent data on contrast-enhanced MDCT in patients suffering from AS have also shown that the use of contrast-enhanced MDCT allows the quantification of non-calcified AS tissue. There is evidence to suggest that such non-calcified, e.g., fibrotic tissue may play a significant role in AS pathophysiology by, for example, impairing leaflet mobility and preventing valve opening. Taking non-calcified tissue into consideration has been proven to increase the predictive value of MDCT in the detection of severe AS and may also help in the determination of borderline-severe AS [33,34].

Notwithstanding, it is important to realize that besides the diagnostic relevance of MDCT, it also possesses proven prognostic relevance. High amounts of AVC are associated with an increased mortality rate in patients with severe AS [12,13]. Therefore, there is an increased interest in AVC determination and a reliable determination of AVC is essential for adequate patient care.

Some limitations need to be addressed. Firstly, our results are not displayed as Agatston Units due to contrast-enhanced MDCT modality. However, the results in mm^3^ are comparable and the results are reproducible and interchangeable [21,22,33,35]. Furthermore, the Agatston Score methodology has been previously validated by Eberhard et al., and our data now suggests that an accurate quantification may also be obtained from contrast-enhanced scans, with the potential to further limit radiation exposure during TAVR planning. Secondly, due to a lack of reference values for contrast-enhanced MDCT-derived AVC in mm^3^, we are not able to report on the potential over- or underestimation of severe aortic stenosis by the software solutions; however, our data show excellent intra- and interobserver variability and therefore we expect a similar risk of under- or overestimation of severe aortic stenosis as compared to the Agatston score method. Thirdly, all data were generated by one MDCT in order to rule out any influence from different MDCT vendors in this study. Therefore, further data for different MDCT scanners would also be desirable.

## 5. Conclusions

Excellent intra- and inter-observer reproducibility, as well as interchangeability, of contrast-enhanced MDCT-derived AVC quantification were demonstrated in this study, independently of the software vendor used. Therefore, the already commercially available software types investigated in this study can be used reliably in clinical practice in order to accurately characterize patients with severe AS.

## Figures and Tables

**Table 1:** The patients’ demographic and baseline characteristics.

Patients	*n* = 50
Demographics	
Age (years)	78 ± 6 (61–92)
Male	32 (64%)
BMI (kg/m^2^)	28 ± 5 (20.7–42,25)
Echocardiographic parameters	
Aortic valve	
V max (m/s)	4.1 ± 0.60 (2.9–5.5)
*P* mean (mmHg)	40 ± 14 (16–68))
AVA VTI (cm^2^)	0.73 ± 0.16 (0.4–1.0)
SVI (ml/m^2^)	36 ± 9 (18–57)
Left ventricle	
LVEF (%)	50.3 ± 11.2 (17.1–68.2)
LVEDD (mm)	45.5 ± 8.7 (29–75)
Comorbidities	
HT	43 (86%)
AF	24 (14.8%)
DM	12 (24%)
CAD	40 (80%)
COPD	6 (12%)

Continuous variables are expressed as mean ± standard deviation. Categorical variables are expressed as absolute value and percentage. BMI: body mass index; V max: peak transvalvular velocity; P mean: mean transvalvular pressure gradient; AVA VTI: aortic valve area based on velocity time integral; SVI: stroke volume index; LVEF: left ventricular function; LVEDD: Left ventricular end-diastolic diameter; HT: hypertension; AF: atrial fibrillation; DM: diabetes mellitus; CAD: coronary artery disease; COPD: chronic obstructive pulmonary disease.

**Table 2:** Inter-vendor agreement and reproducibility.

		3 Mensio–CVI 42			CVI 42–Syngo.Via			3 Mensio–Syngo.Via		
		Mean Difference (SD)	ICC (95%CI)	CoV (%)	Mean Difference (SD)	ICC (95%CI)	CoV (%)	Mean Difference (SD)	ICC (95%CI)	CoV (%)
Inter-vendor	AVC (mm^3^)	−0.06 (84.16)	0.996 (0.992–0.998)	9	−7 (68.60)	0.997 (0.995–0.998)	7.3	−7.06 (115.07)	0.992 (0.986–0.995)	12.2

The inter-vendor agreement and reproducibility with regards to the degree of AVC. The results are reported as mean ± standard deviation (SD). AVC: aortic valve calcification; ICC: intraclass-correlation coefficient; CoV: coefficient of variation; SD: standard deviation; CI: confidence interval.

**Table 3:** Intra- and inter-observer agreement and reproducibility.

		3 Mensio			CVI 42			Syngo.Via		
		Mean Difference (SD)	ICC (95%CI)	CoV (%)	Mean Difference (SD)	ICC (95%CI)	CoV (%)	Mean Difference (SD)	ICC (95%CI)	CoV (%)
Intra-observer	AVC (mm^3^)	−19.28 (45.07)	0.999 (0.995–1.000)	3.9	−10.28 (18.6)	1.000 (0.999–1.000)	1.6	−24.81 (48.52)	0.998 (0.993–1.000)	4.1
Inter-observer	AVC (mm^3^)	−7.14 (16.20)	1.000 (0.999–1.000)	1.4	1.74 (11.83)	1.000 (1.000–1.000)	1.0	0.65 (79.43)	0.996 (0.985–0.999)	6.7

The intra- and inter-observer agreement and reproducibility with regards to the degree of AVC, based on a repeated measurement of a subset of 10 randomly selected patients. Results are reported as mean difference ± standard deviation (SD). AVC: aortic valve calcification; ICC: intraclass-correlation coefficient; CoV: coefficient of variation; SD: standard deviation; CI: confidence interval.

## Data Availability

All data that support the findings of this study are available upon request from the corresponding author.
